# Supplementary material for: Does Creatine Supplementation Enhance Performance in Active Females? A Systematic Review
Source: Nutrients. 2025 Jan 10;17(2):238. doi: 10.3390/nu17020238 (PMC11767391; doi:10.3390/nu17020238)
Supplement: Supplementary file 1 [file nutrients-17-00238-s001.zip › nutrients-3397314-supplementary.pdf]

Does creatine supplementation enhance performance in active females? A systematic literature review.

Ryan Tam[1], Lachlan Mitchell[2], Adrienne Forsyth[3]

1. Discipline of Nutrition and Dietetics, School of Behavioural and Health Sciences, Australian Catholic University, Blacktown, Australia
2. Discipline of Nutrition and Dietetics, School of Behavioural and Health Sciences, Australian Catholic University, North Sydney, Australia
3. Discipline of Nutrition and Dietetics, School of Behavioural and Health Sciences, Australian Catholic University, Melbourne, Australia

Corresponding author: Ryan Tam ryan.tam@acu.edu.au

Supplementary Figure S1. Full search strategy by database

Cinahl:<br>creatine AND (woman OR women OR female) AND (exercise OR performance OR endurance OR aerobic OR Strength OR power OR anaerobic OR physique OR Body composition)<br>**Expanders** - Apply equivalent subjects<br>**Search modes** - Find all my search terms
PubMed: Search:<br>**((creatine) AND (woman OR women OR female)) AND (exercise OR performance OR endurance OR aerobic OR Strength OR power OR anaerobic OR physique OR body composition)**<br>("creatine"[MeSH Terms] OR "creatine"[All Fields] OR "creatin"[All Fields] OR "creatines"[All Fields]) AND ("womans"[All Fields] OR "women"[MeSH Terms] OR "women"[All Fields] OR "woman"[All Fields] OR "women s"[All Fields] OR "womens"[All Fields] OR ("womans"[All Fields] OR "women"[MeSH Terms] OR "women"[All Fields] OR "woman"[All Fields] OR "women s"[All Fields] OR "womens"[All Fields]) OR ("femal"[All Fields] OR "female"[MeSH Terms] OR "female"[All Fields] OR "females"[All Fields] OR "female s"[All Fields] OR "femals"[All Fields])) AND ("exercise"[MeSH Terms] OR "exercise"[All Fields] OR "exercises"[All Fields] OR "exercise therapy"[MeSH Terms] OR ("exercise"[All Fields] AND "therapy"[All Fields]) OR "exercise therapy"[All Fields] OR "exercising"[All Fields] OR "exercise s"[All Fields] OR "exercised"[All Fields] OR "exerciser"[All Fields] OR "exercisers"[All Fields] OR ("perform"[All Fields] OR "performable"[All Fields] OR "performance"[All Fields] OR "performance s"[All Fields] OR "performances"[All Field]
Scopus:<br>SCOPUS - (TITLE (creatine) AND TITLE-ABS-KEY (woman OR women OR female) AND TITLE-ABS-KEY<br>(exercise OR performance OR endurance OR aerobic OR strength OR power OR anaerobic OR physique O R "body composition"))
SPORTDiscus:<br>creatine AND (woman OR women OR female) AND (exercise OR performance OR endurance OR aerobic OR Strength OR power OR anaerobic OR physique OR Body composition)<br>**Expanders** - Apply equivalent subjects

**Search modes** - Find all my search terms
Web of Science:<br><br>((ALL=(creatine)) AND ALL=(woman OR women OR female)) AND ALL=(exercise OR performance OR endurance OR aerobic OR Strength OR power OR anaerobic OR physique OR "Body composition")
